# Cortical Pyramidal and Parvalbumin Cells Exhibit Distinct Spatiotemporal Extracellular Electric Potentials

**DOI:** 10.1523/ENEURO.0176-23.2023

**Published:** 2023-07-04

**Authors:** Lihao Guo

**Affiliations:** Division of Computational Science and Technology, KTH Royal Institute of Technology, 100 44 Stockholm, Sweden

## Abstract

**Highlighted Research Paper:** L. J. Sukman and E. Stark, “Cortical pyramidal and parvalbumin cells exhibit distinct spatiotemporal extracellular electric potentials.” *eNeuro* (2022).

The classification of different types of neurons is a central problem in neuroscience, and previous research has yielded much knowledge about their firing properties, morphologic features, connectivity patterns, and genetic expressions. Recent studies aim to integrate these characteristics to provide a more unified understanding of neuron types, a synthesized fingerprint for each neuron type. Therefore, understanding the relationship between these different characteristics, such as how different morphologies give rise to different firing properties, is increasingly important.

Direct measurements of signal transfer in axons and dendrites would be ideal for answering these questions, but measuring a large number of neurons is difficult, if not impossible. An alternative approach is to use extracellular recordings of voltage potential, which is induced by spikes. Computational models that attempt to explain the extracellular waveform generated by spikes date back to Gold et al. (2006), who suggested that spikes induce an extracellular spatiotemporal distribution of voltage potential because of morphology. The relative position of the electrode to the neuron plays an important role in measuring the spike waveform.

To experimentally investigate the spatiotemporal features of extracellular waveform from spiking activities, researchers need to go beyond traditional experimental design. Lior J. Sukman and Eran Stark (Sukman and Stark, 2022) were able to measure the extracellular distribution of voltage potential from the hippocampal region CA1 and neocortex of freely moving mice in vivo by orienting the multichannel electrode parallel to the dendritic tree of pyramidal cells (PYR) and parvalbumin-immunoreactive (PV) cells. Ground truth of cell types are obtained with optogenetic tagging. Compared with the traditional analysis of spike waveform, such extracellular recordings contain spatiotemporal information because of morphology and inputs, which are known to be different for PYR and PV cells (Zeng and Sanes, 2017). This poses two questions. First, can neurons, such as PYR and PV cells, be classified based solely on spatiotemporal features derived from these multichannel recordings? Second, what additional information can these spatiotemporal features provide?

Consistent with previous studies, PYR and PV cells could be almost perfectly classified using the waveform information. By focusing on the strongest channel of extracellular recording, the shape of the spike was used to classify the neurons. To determine if there is unique information in the spatiotemporal distribution, the waveform information was then removed with an event-based δ-transformation, leaving only the spike timing distribution of extracellular voltage potential. The transformed data could be used to classify PYR and PV accurately using a simple random forest model. Compared with PV cell spikes, PYR spikes exhibited higher spatial synchrony at the beginning of the extracellular spike and lower synchrony at the trough. The results support the hypothesis that spatiotemporal information because of morphology and input contains a unique fingerprint for different types of neurons.

Apart from achievable classification of neuron types, spatiotemporal features capture more information than waveform alone. Comparing classifiers trained using waveform features on neocortical neurons to those using spatiotemporal features, the former could generalize to CA1 neurons very well, whereas the latter could only generalize with lower accuracy. This indicates that the spike waveform contains a more intrinsic signature of each neuron type, while spatiotemporal distribution contains information about the neuron's embedding in a network. This raises an interesting question for computational neuroscientists: how important is the intrinsic fingerprint of each neuron type compared with their embedding in a network with regard to functions?

To better address the question, a more careful feature extraction is necessary. The authors currently rely on statistical averaged descriptors, placing emphasis on their independence to ensure orthogonality. However, since spatiotemporal information is contained in the joint distribution of multiple channels, it is possible that the underlying factors may not have biological interpretations at all when decomposed into independent ones. Instead of arbitrarily designed features, one alternative is to employ a nonlinear dimensionality reduction of the multichannel recordings to obtain a low-dimensional representation (McInnes et al., 2018). Classification based on such representation could potentially disentangle not only the intrinsic neuron type but also the embedding in a network, such as in neocortex or CA1, from the extracellular recordings of spiking activities.

This study provides solid experimental support for an association between morphologic features, connectivity patterns, and firing properties. Additionally, categorization on neuron subtypes would benefit from using spatiotemporal distribution of extracellular spike waves where the waveform alone is limited. It would deepen our understanding of neuron types to expand the experimental design by incorporating input statistics, e.g., examining extracellular neuronal activities in other states besides freely moving cases such as in stimulus-evoked state where firing statistics can vary substantially (Churchland et al., 2010).

## Synthesis

Reviewing Editor: Arvind Kumar, KTH Royal Institute of Technology
